# Immunogenicity and protective efficacy of enterotoxigenic *Escherichia coli* (ETEC) total RNA against ETEC challenge in a mouse model

**DOI:** 10.1038/s41598-020-77551-8

**Published:** 2020-11-25

**Authors:** Mandi Liu, Yue Zhang, Di Zhang, Yun Bai, Guomei Liu, Pei Li, Jianguo Li, Yan Li

**Affiliations:** 1 College of Veterinary Medicine, Hebei Agricultural University, Baoding, 071000 Hebei China; 2 Department of Biochemistry and Molecular Biology, Hebei Key Laboratory of Chinese Medicine Research on Cardio-Cerebrovascular Disease, Hebei University of Chinese Medicine, Shijiazhuang, 050200 Hebei China; 3 College of Animal Science and Technology, Hebei Agricultural University, Baoding, 071000 Hebei China

**Keywords:** Bacterial host response, Antimicrobial responses, Vaccines

## Abstract

Enterotoxigenic *Escherichia coli* (ETEC), an essential cause of post-weaning diarrhea (PWD) in piglets, leads to significant economic losses to the pig industry. The present study aims to identify the role of ETEC total RNA in eliciting immune responses to protect animals against ETEC infection. The results showed that the total RNA isolated from pig-derived ETEC K88ac strain effectively stimulated the IL-1β secretion of porcine intestinal epithelial cells (IPEC-J2). The mouse model immunized with ETEC total RNA via intramuscular injection (IM) or oral route (OR) was used to evaluate the protective efficiency of the ETEC total RNA. The results suggested that 70 μg ETEC total RNA administered by either route significantly promoted the production of the serum IL-1β and K88ac specific immunoglobulins (IgG, IgM, and IgA). Besides, the ETEC RNA administration augmented strong mucosal immunity by elevating K88ac specific IgA level in the intestinal fluid. Intramuscularly administered RNA induced a Th1/Th2 shift toward a Th2 response, while the orally administered RNA did not. The ETEC total RNA efficiently protected the animals against the ETEC challenge either by itself or as an adjuvant. The histology characterization of the small intestines also suggested the ETEC RNA administration protected the small intestinal structure against the ETEC infection. Particularly of note was that the immunity level and protective efficacy caused by ETEC RNA were dose-dependent. These findings will help understand the role of bacterial RNA in eliciting immune responses, and benefit the development of RNA-based vaccines or adjuvants.

## Introduction

Enterotoxigenic *Escherichia coli* (ETEC) is one of the main pathogens that cause post-weaning diarrhea (PWD) in piglets^1^. PWD is endemic and highly infectious in piglets. It typically occurs within 2 weeks after weaning and is characterized by diarrhea, dehydration, and even death^2,3^. The resulting weight loss, secondary infection, and mortality cause significant economic loss to the global pig industry. However, the most commonly applied control strategies involving antibiotics and ZnO have been restricted in many countries due to the induction of antimicrobial resistance and environmental damage^4–6^. Vaccination has become the dominant and most effective way to protect piglets against PWD. At present, antitoxin vaccines are mainly developed using virulence factors, including enterotoxin or fimbriae as antigens^7–10^. ETEC strains causing porcine diarrhea include five different fimbrial subtypes, namely K88 (F4), K99 (F5), F41, F18 and 987P (F6). Enterotoxins may be classified into heat-labile toxins (LT) and heat-stable toxins (ST)^11^. Due to the mixed infection of various ETEC fimbriae, the current vaccination strategies are designed to express multiple enterotoxins or fimbriae subtypes. Therefore, it will be beneficial to discover novel antigens that may improve the immunogenicity and effectiveness of the vaccines.

Pathogen structural components such as lipopolysaccharide (LPS), DNA and RNA, are characterized as pathogen-associated molecular patterns (PAMPs). Pattern recognition receptors (PRRs) of the host cells can identify the PAMPs and trigger specific cytokine responses. As an important component of PAMPs, pathogen RNA can elicit the innate immunity in many host cells through the recognition by various PRRs^12,13^. For example, porcine reproductive and respiratory syndrome virus (PRRSV) RNA, recognized by host helicase DDX19A, stimulates IL-18 and IL-1β secretion in porcine alveolar macrophages through the activation of the NLRP3 pathway^14^. In addition, non-pathogenic *E. coli* RNA promotes the secretion of IL-18 and IL-1β in human macrophages, depending on the recognition by DHX33 and subsequent activation of NLRP3 inflammasome^15^. Aside from innate immunity, pathogen RNA also plays a role as an antigen to elicits adaptive immunity. When supplemented as a vaccine adjuvant, the Sendai virus-derived RNA, a RIG-I agonist, was shown to have significantly promoted the survival rate of the mice immunized with an influenza A virus vaccine against influenza A challenge^16^. Sander et al. discovered that the total RNA derived from non-pathogenic *E. coli* DH5α is necessary for heat-inactivated *E. coli* to trigger humoral immunity in mice^17^. The above studies indicated that both viral and bacterial RNA could be recognized as antigens by the PRRs of the host cells, thereby inducing both innate and adaptive immunities.

Although non-pathogenic *E. coli* RNA is capable to induce immune responses, whether or not the diarrheagenic ETEC RNA is immunostimulatory has yet to be investigated. In this study, we found that the total RNA from pig-derived ETEC K88ac strain stimulated IL-1β production of porcine intestinal epithelial cells (IPEC-J2). Therefore, we propose that the ETEC total RNA might stimulate humoral immunity and protect the animals against ETEC infection. For this purpose, BALB/c mice were administrated with the ETEC total RNA intramuscularly or orally. We examined the humoral and mucosal immunity by detecting the K88ac specific immunoglobulin levels. The protective efficiency against the ETEC challenge was also evaluated via the survival rate and weight loss. Finally, in what manner RNA immunization protected the small intestine was assessed by the histology characterization and expression levels of tight junction proteins. The results demonstrated that ETEC total RNA confers immunogenicity and effectively protects against ETEC infection in the mouse model.

## Results

### ETEC total RNA induces IL-1β secretion of porcine intestinal epithelial cells

**Figure 1:**
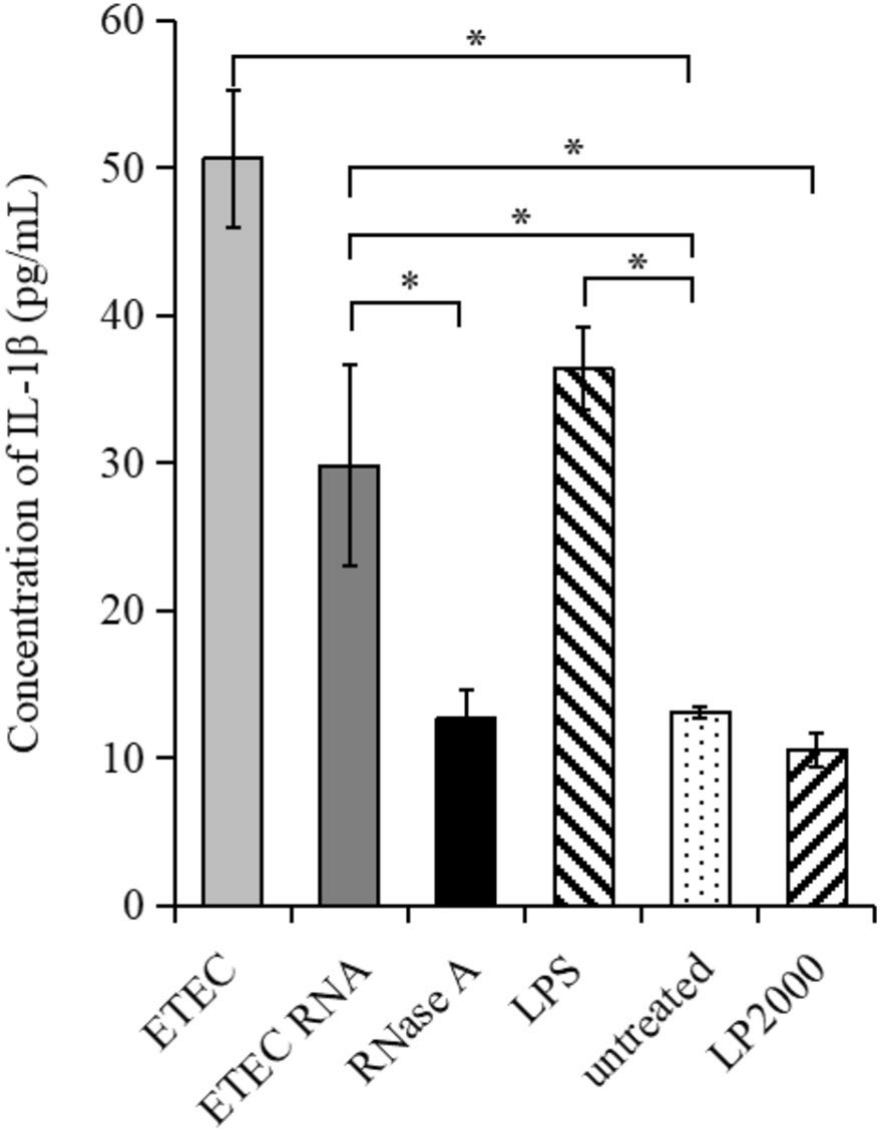
IL-1β secretion levels of IPEC cells infected by ETEC bacteria and stimulated by its total RNA. The IPEC-J2 cells were infected with ETEC bacteria at MOI of 400 for 24 h. For RNA stimulation, the IPEC cells were transfected with ETEC total RNA via lipofectamine 2000 (LP2000). RNase A indicates the cells were transfected with RNases treated ETEC total RNA. For the positive control, lipopolysaccharide (LPS) was used to stimulate IPEC cells for 12 h, followed by 5 mM ATP incubation for an additional 5 h. Untreated IPEC-J2 cells and the cells transfected only with LP2000 were used as the negative controls. The y-axis displays the medium concentration of IL-1β secreted by the IPEC cells. The error bars represent the standard deviations from three replicates. *P < 0.05.

The ETEC K88ac infection and the ETEC total RNA transfection significantly induced the IL-1β secretion of IPEC-J2 cells (Fig. 1). To exclude the possibility that the contaminants from the RNA isolation caused the IL-1β responses, we treated ETEC total RNA with RNase A before transfection. In this case, the IL-1β level was as low as the untreated cells, confirming the RNA is responsible for the stimulation. The IL-1β response was also eliminated when the cells were only treated with transfection reagent. It excluded the possibility that the IL-1β stimulation was due to transfectamine. Therefore, it was suggested that the ETEC total RNA was able to induce IL-1β production of IPEC-J2 cells.

### ETEC total RNA immunization triggers serum IL-1β production in the mouse model

**Figure 2:**
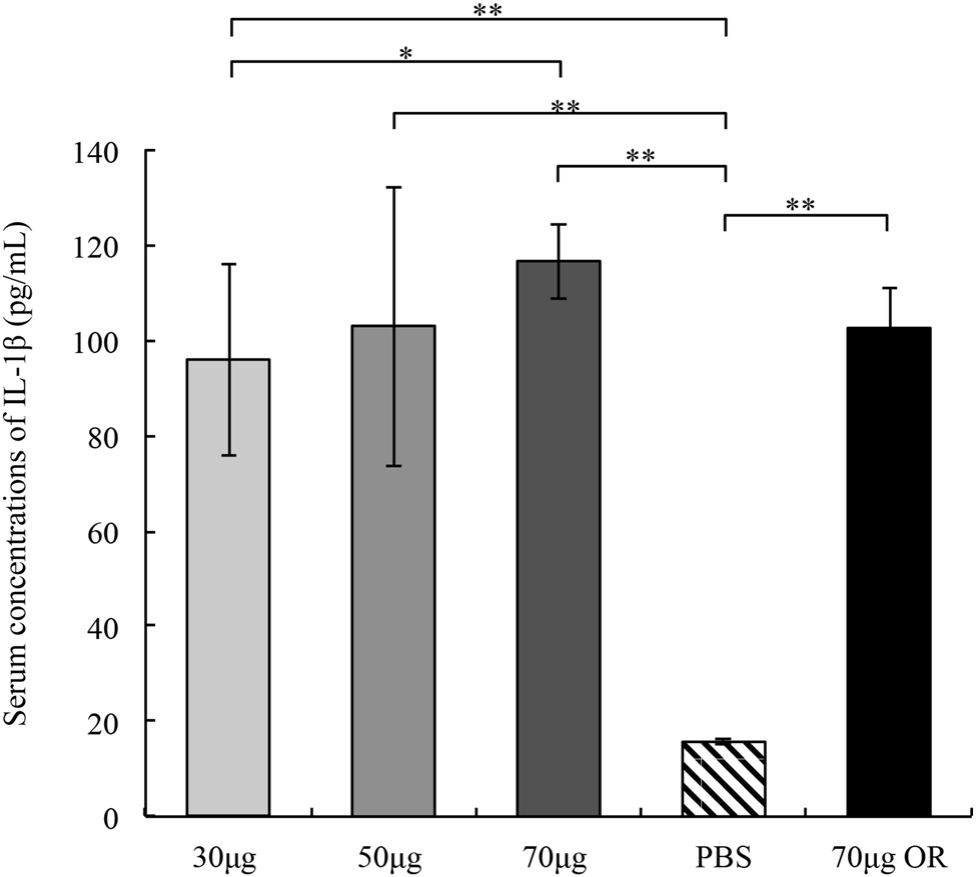
Serum IL-1β levels of ETEC total RNA immunized mice. Four groups of mice (n = 8) were intramuscularly immunized with PBS, 30 μg, 50 μg, and 70 μg ETEC total RNA, respectively. The fifth group of mice was orally immunized with 70 μg ETEC total RNA. The serum IL-1β levels were detected at 20 dpi. The error bars represent the standard deviations from eight mice. **P < 0.01, *P < 0.05.

To investigate whether or not the ETEC total RNA also induces the IL-1β responses in the animal model, we assessed the serum IL-1β level in the mice immunized with ETEC total RNA at 20 days post-immunization (dpi) using ELISA. As shown in Fig. 2, the RNA delivered via the intramuscular route (IM) or oral route (OR) both significantly increased serum IL-1β level. While the mice that orally immunized with 70 μg ETEC total RNA exhibited a lower IL-1β response than the mice intramuscularly administrated with 70 μg ETEC total RNA. Besides, the average serum IL-1β levels in the 30 μg, 50 μg, and 70 μg ETEC RNA intramuscularly administrated groups were 95.93 pg/mL, 103.02 pg/mL, and 116.64 pg/mL, respectively. All of them were significantly higher than the IL-1β level in the PBS administrated control group (15.72 pg/mL) (*P* < 0.01). The average serum IL-1β concentration in the 70 μg RNA intramuscularly immunized mice was slightly higher and significantly higher than that in the 50 μg and 30 μg RNA immunized mice, respectively. Thus, the serum IL-1β production was positively correlated with the immunization dosage of ETEC total RNA.

### ETEC total RNA immunization promotes the synthesis of ETEC K88ac specific immunoglobulins in the mouse model

**Figure 3:**
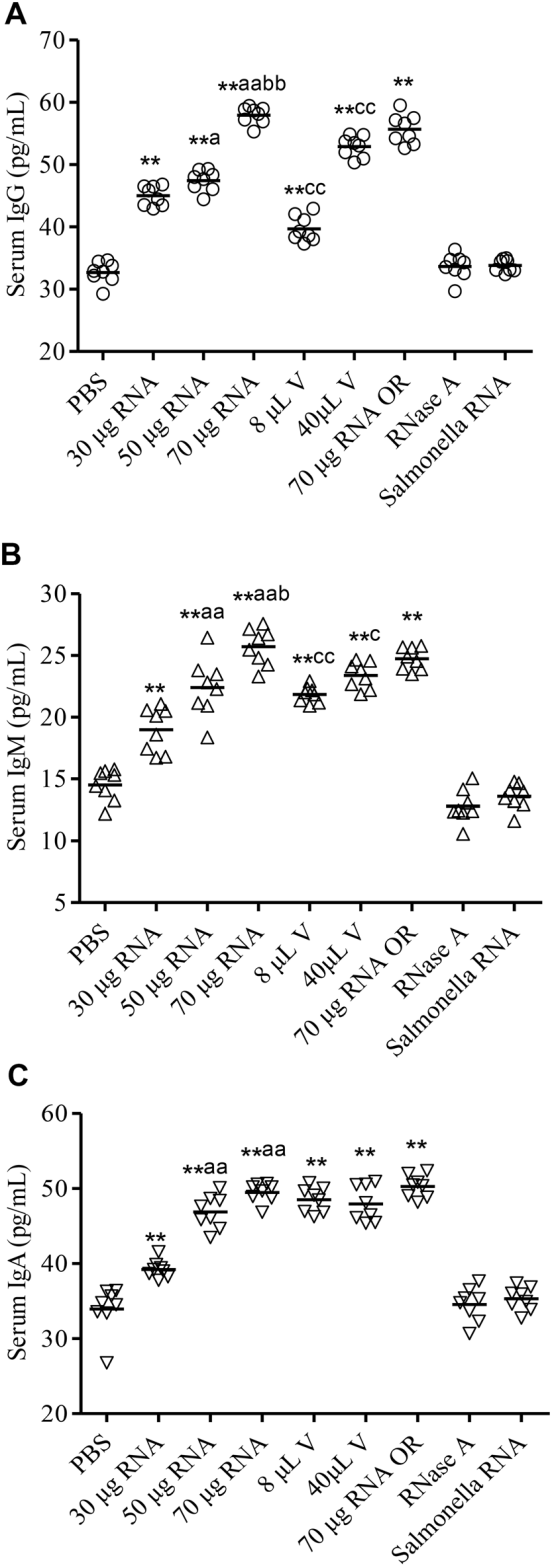
ETEC K88ac specific immunoglobulin levels in the serum of the immunized mice. The eight experimental groups of mice (n = 8) were intramuscularly injected with PBS, ETEC total RNA (30 μg RNA; 50 μg RNA; 70 μg RNA), ETEC vaccine (8 μL V; 40 μL V), RNase A treated ETEC RNA (labeled as RNase A) and 70 μg *Salmonella* RNA in a total volume of 100 μL. The ninth group of mice (n = 8) was orally immunized with 70 μg ETEC total RNA. The serum concentrations of IgG (**A**), IgM (**B**), and IgA (**C**) were measured at 20 dpi using ELISA. **P < 0.01 compared with the PBS control group. ^aa^*P* < 0.01, ^a^*P* < 0.05, compared with the 30 μg ETEC RNA administrated mice. ^bb^*P* < 0.01, ^b^*P* < 0.05 compared with the 50 μg ETEC RNA administrated mice. ^cc^*P* < 0.01, ^c^*P* < 0.05 compared with the 70 μg ETEC RNA administrated mice.

Next, we assessed the serum levels of ETEC K88ac specific immunoglobulins to investigate whether or not RNA immunization induces adaptive immune responses. Three groups of mice (n = 8) were respectively immunized with 30 μg, 50 μg, and 70 μg ETEC total RNA via IM. The vaccine administrated groups performed as a positive control, while the PBS group acted as a negative control. A recommended dosage (40 μL) and a suboptimal dosage (8 μL) of the vaccine were administered to the animals via IM, respectively. The serum immunoglobulin levels were measured at 20 dpi using ELISA. The average concentrations of ETEC k88ac specific immunoglobulins (IgG, IgA, and IgM) in the ETEC RNA and the vaccine immunized mice were all significantly higher than those in the PBS treated mice (*P* < 0.01) (Fig. 3A–C). With the increase of the RNA immunization dosage from 30 μg, 50 μg to 70 μg, the serum levels of IgG and IgM also significantly increased (Fig. 3A, B). The IgA level of the 50 μg RNA immunization group was considerably higher than that of the 30 μg immunization group (*P* < 0.01), while the 70 μg RNA administration caused a slightly higher IgA response than the 50 μg RNA administration (Fig. 3C). Therefore, the immunity level in the mouse model was shown to be positively correlated with the immunization dosage of ETEC total RNA. In comparison with the ETEC vaccine, 70 μg ETEC RNA triggered considerably higher levels of IgG and IgM. No obvious difference was observed in stimulating serum IgA responses between the immunization with 70 μg ETEC RNA and the vaccines. In order to explore the effects of RNA delivery routes, 70 μg ETEC RNA was administered to another group of mice via OR. In this case, we detected virtually equivalent levels of the specific immunoglobulins from the mice immunized via IM and OR, indicating the RNA was able to be effectively delivered by both routes (Fig. 3A–C). In order to eliminate the possibility that the immune responses in RNA administrated groups were due to the contaminants during the RNA isolation process, 70 μg ETEC RNA was treated with RNase before intramuscular injection. It shows that the serum immunoglobulins production was completely removed after RNase treatment, confirming that the immunity stimulation was dependent on RNA (Fig. 3A–C). The immunoglobulin-inducing activity was not detected in the mice administrated with *Salmonella* RNA, which proved that ETEC K88ac immunoglobulins were specifically stimulated by the ETEC RNA (Fig. 3A–C).

### ETEC total RNA induces a shift in the Th1/Th2 cytokine balance

To detect whether ETEC RNA immunization affects Th1 and Th2 cytokine production, we measured the serum levels of Th1 cytokine IFN-γ and Th2 cytokine IL-4 by ELISA at 20 dpi (Table 1). Compared to the PBS control group, immunization with 70 μg ETEC total RNA via IM significantly elevated the IFN-γ and IL-4 levels (*P* < 0.05 and *P* < 0.05). The IFN-γ/IL-4 ratio in the RNA administrated group via IM was significantly lower than that from the control group (*P* < 0.05). It indicates the ETEC RNA influences cytokine production, resulting in a shift favoring a Th2 response. Interestingly, in animals immunized with the same amount of ETEC RNA via OR, the IFN-γ/IL-4 ratio was slightly higher than the control group. Thus, the RNA delivery route affects the Th1/Th2 cytokine balance differently. A considerably lower IL-4 level was detected in the ETEC vaccine administrated group at 20 dpi. Therefore, its IFN-γ/IL-4 ratio was significantly higher than the control group (*P* < 0.01), indicating the inactivated ETEC vaccine induced a stronger Th1 response.

**Table 1:** Expression of IFN-γ and IL-4 in serum from the administrated mice.

Group	n	INF-γ (pg/mL)	IL-4 (pg/mL)	INF-γ/IL4 ratio
PBS	8	133.69 ± 30.46	72.35 ± 7.23	1.85 ± 0.23
ETEC RNA (IM)	8	171.83 ± 34.43*	112.93 ± 37.23*	1.52 ± 0.19*
Vaccine	8	103.65 ± 31.41	17.91 ± 4.05**	4.05 ± 0.44**
ETEC RNA (OR)	8	248.52 ± 28.85**	119.87 ± 5.78**	2.08 ± 0.14

Values are means ± SD for eight mice in one group.

*P < 0.05 in comparison with the PBS group.

**P < 0.01 in comparison with the PBS group.

### ETEC total RNA immunization induces intestinal mucosal immunity

**Figure 4:**
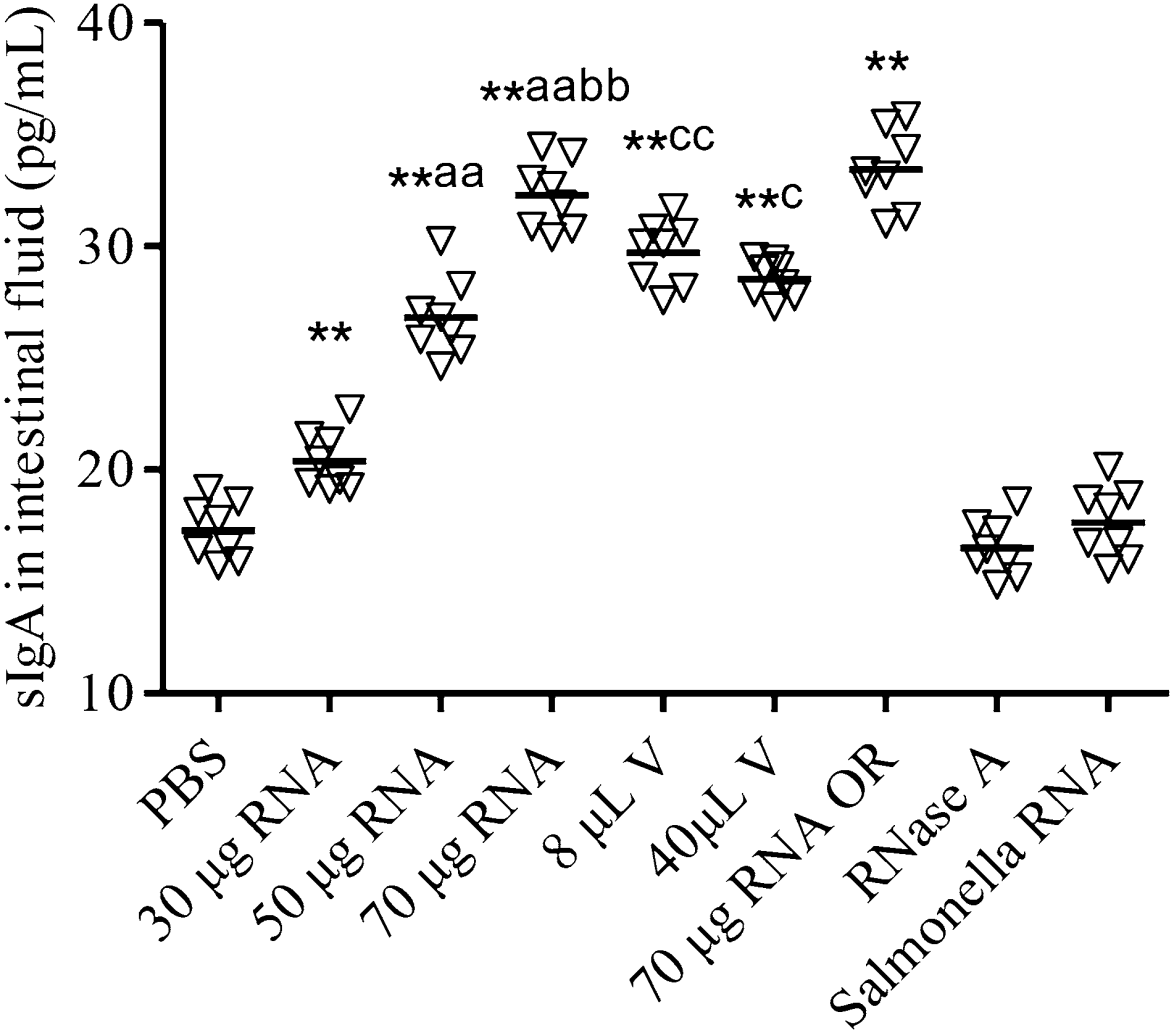
ETEC K88ac specific sIgA levels in the intestinal fluid of the administrated mice. The eight experimental groups of mice (n = 8) were intramuscularly injected with PBS, ETEC total RNA (30 μg RNA; 50 μg RNA; 70 μg RNA), ETEC vaccine (8 μL V; 40 μL V), RNase A treated ETEC RNA (labeled as RNase A) and 70 μg *Salmonella* RNA in a total volume of 100 μL. The ninth group of mice (n = 8) was orally immunized with 70 μg ETEC total RNA. The intestinal fluids were collected at 20 dpi, and the ETEC K88ac specific IgA levels were assessed using ELISA. **P < 0.01 compared with the PBS control group. ^aa^*P* < 0.01 compared with the 30 μg ETEC RNA administrated mice. ^bb^*P* < 0.01 compared with the 50 μg ETEC RNA administrated mice. ^cc^*P* < 0.01, ^c^*P* < 0.05 compared with the 70 μg ETEC RNA administrated mice.

To assess mucosal immune response, we examined ETEC K88ac specific IgA levels in the intestinal fluid from all the administrated groups at 20 dpi using ELISA (Fig. 4). The RNA immunized groups (30 μg, 50 μg, 70 μg) showed clearly higher IgA production than the PBS control group in an RNA dose-dependent manner. Again, we could not detect the intestinal mucosal antibodies in the RNase A treated samples and *Salmonella* RNA administrated samples, indicating the mucosal immune responses were dependent on the ETEC RNA.

### ETEC total RNA immunization confers protection against ETEC challenge

**Figure 5:**
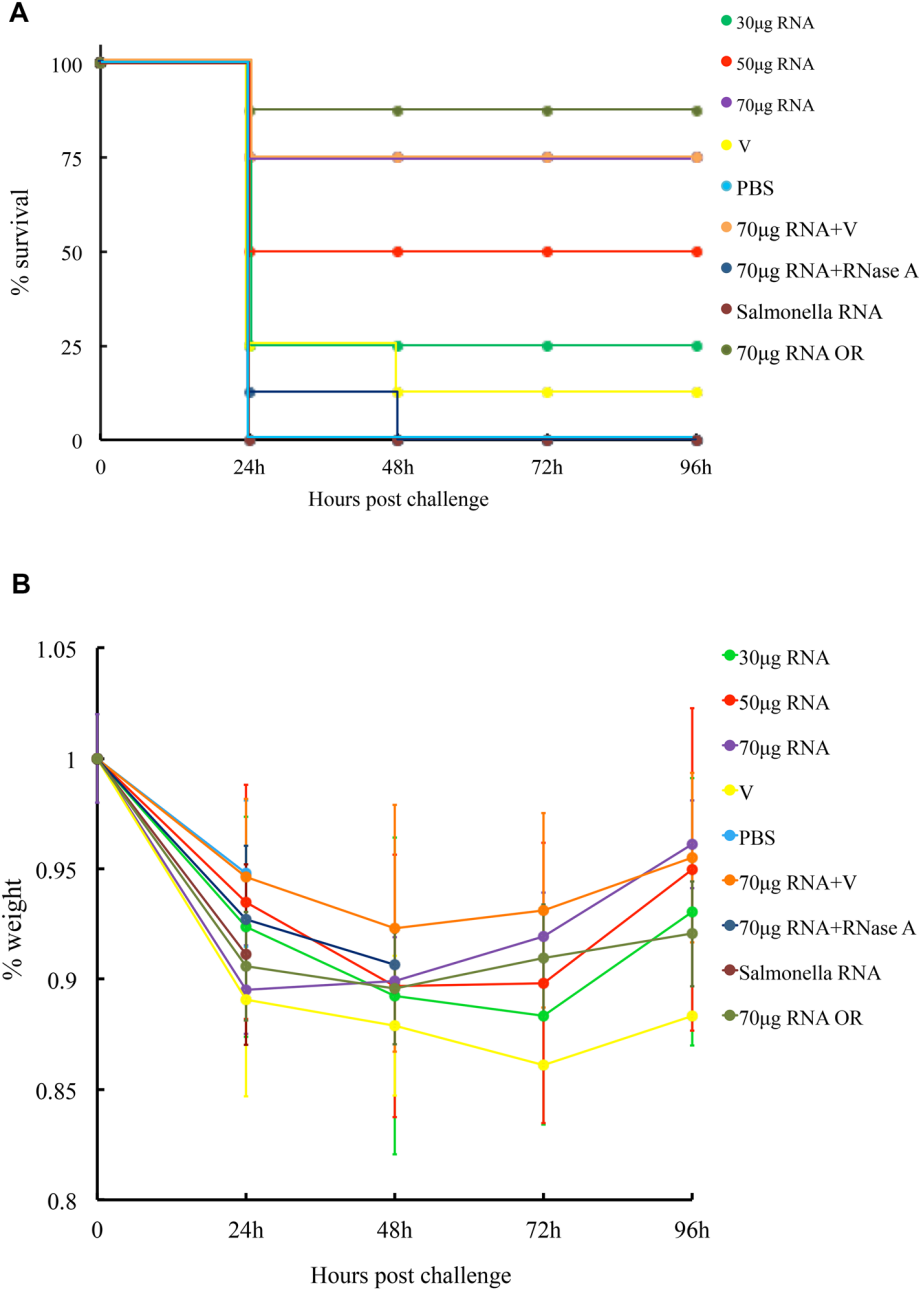
Survival rate (**A**) and weight maintenance rate (**B**) of the immunized mice following the ETEC challenge. The eight groups of mice (n = 8) were intramuscularly injected with ETEC total RNA (30 μg RNA; 50 μg RNA; 70 μg RNA), 8 μL ETEC vaccine (V), PBS, 8 μL vaccine plus 70 μg ETEC RNA (70 μg RNA + V), RNase A treated ETEC RNA (70 μg RNA + RNase A) and 70 μg *Salmonella* RNA in a total volume of 100 μL. The ninth group of mice (n = 8) was orally immunized with 70 μg ETEC total RNA. Body weight maintenance was calculated using actual body weight at different time points post-challenge divided by the initial body weight. The error bars in panel B represent the standard deviations from the mice that survived. No deviation is shown at the time point when only one mouse was alive.

To evaluate whether or not the immune responses elicited by ETEC total RNA could protect the animals, we carried out a lethal challenge experiment. In this case, eight groups of mice (n = 8) were immunized with ETEC vaccine (8 μL), ETEC total RNA (30 μg, 50 μg, 70 μg), vaccine/RNA mixture (8 μl vaccine + 70 μg ETEC total RNA), PBS, ETEC total RNA treated with RNase A, and *Salmonella* RNA (70 μg). In order to clearly reflect the protective function of the ETEC RNA as an adjuvant, an 8-μL dose of vaccine was used to co-administrate the mice with 70 μg ETEC total RNA. A ninth group of mice was orally immunized with 70 μg ETEC total RNA to compare the effects of RNA delivery routes. At 23 dpi, the mice were challenged with 3 × 10^8^ CFU of ETEC K88ac bacteria, and the deaths and average body weight of each group were recorded within 96 h post-challenge (Fig. 5). As shown in Fig. 5A, the survival rates of the RNA immunization group and vaccine/RNA mixture immunization group achieved 75%. In comparison, only 12.5% of the mice survived in the vaccine immunization group. All of the animals from the negative controls, including PBS, RNase A treated ETEC RNA, and *Salmonella* RNA administrated groups were dead within 48 h post-challenge. In comparison with the ETEC vaccine alone, supplementing ETEC RNA as an adjuvant reduced the mortality remarkably. It demonstrated that ETEC total RNA induced immunity protected the animals against ETEC infection. The respective survival rates of the 30 μg, 50 μg, and 70 μg RNA immunized groups were 25%, 50%, and 75%, indicating a positive correlation between the dosage of ETEC total RNA immunization and its protective efficacy (Fig. 5A). Besides, 87.5% of the mice orally immunized with 70 μg RNA survived after the ETEC challenge, which is slightly higher than the mice intramuscularly immunized with equivalent RNA.

We also monitored the body weights of the mice within 96 h post-challenge (Fig. 5B). Except for all deaths in the negative control groups, the average body weights of all the immunized groups displayed a rapid decline, followed by a final recovery. The 70 μg RNA or RNA/vaccine mixture immunized group lost an average of 10.5% and 7.7% of the initial weight at the early stage, then recovered to 96.1% and 95.5%. The average weight of the vaccine treated group decreased to 86.1% of the initial weight but only rose to 88.3% at 96 h post-challenge. Therefore, it shows that the ETEC RNA immunization effectively maintained body weight. The ETET RNA treated groups (30 μg, 50 μg, 70 μg) also recovered the average body weight in a dose-dependent manner. Combined with the survival rate analysis, we concluded that ETEC total RNA immunization with an appropriate dose provided effective protection against the ETEC challenge. Supplementing ETEC RNA to the vaccine significantly enhanced its protection effectiveness.

### ETEC total RNA immunization protects the integrity of the small intestinal structure

**Figure 6:**
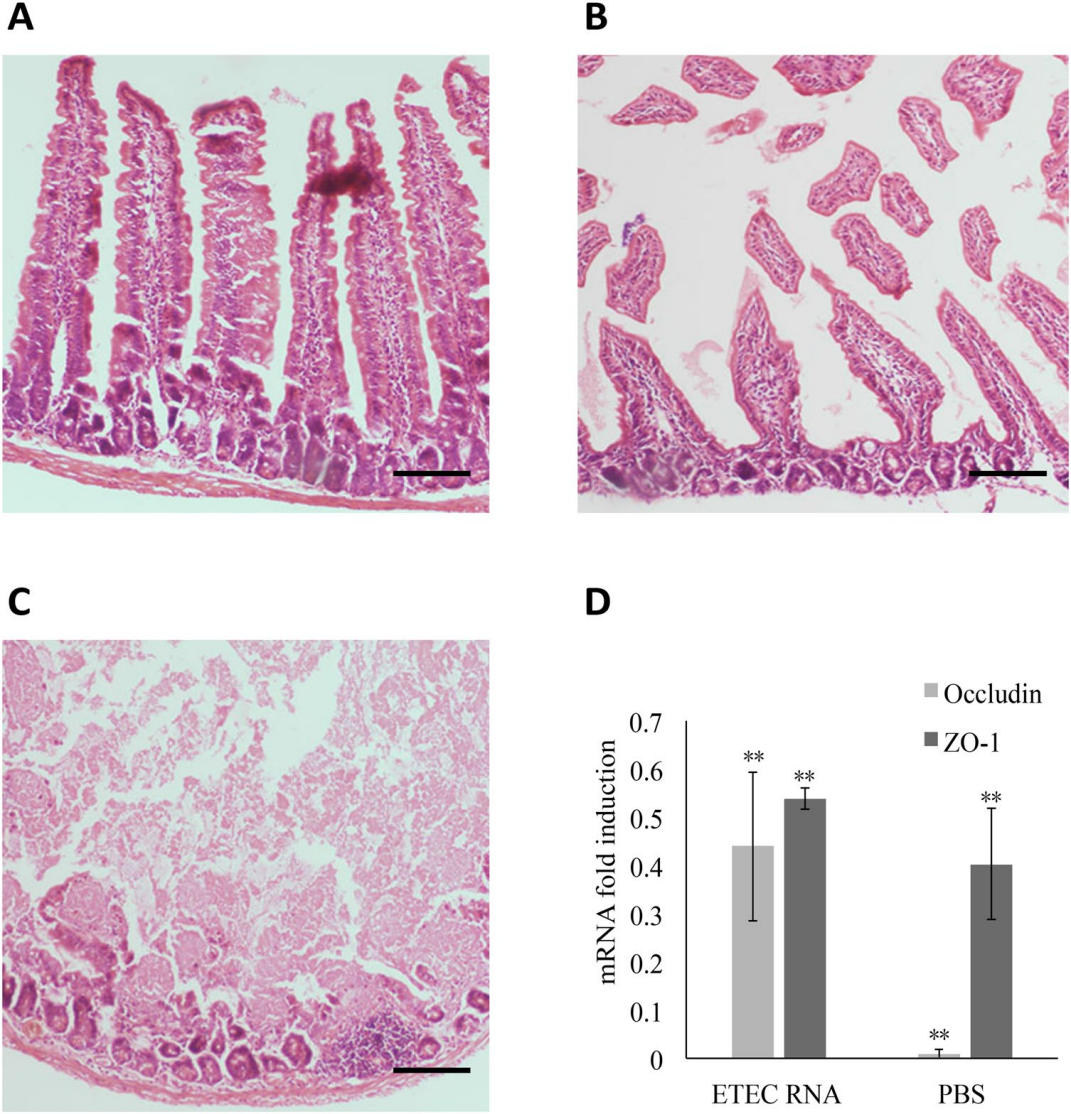
ETEC RNA immunization protected the small intestine against the ETEC challenge. The histology of the small intestines from the unchallenged mice (**A**), ETEC RNA immunized mice post-challenge (**B**), and PBS immunized mice post-challenge (**C**) were observed 5 days post-challenge, respectively. The scale bar represents 50 μm. (**D**) The mRNA expression of the intestinal tight junction proteins from the RNA immunized mice and PBS treated mice. The fold induction of the mRNA from the unchallenged mice was set to 100%. Occludin is indicated by the light grey bar, while the dark grey bar represents ZO-1. The error bars represent the standard deviations from three random mice in each group. **P < 0.01.

To investigate how ETEC RNA immunization protects the physiological structure of the murine small intestine, we observed the histopathological characteristics of the duodenums after the ETEC challenge. In comparison with the unchallenged mice, the small intestinal villi from the RNA immunized mice exhibited shorter finger-like luminal projections with an enlarged crypt region post-challenge (Fig. 6A, B). But the outline of the villi was still discernable. However, the villi from the PBS group were completely damaged post-challenge (Fig. 6C). The results showed that immunization with ETEC total RNA protected the colonic histology of murine small intestine against ETEC infection.

To evaluate in what manner ETEC RNA immunization affects the integrity of the small intestines following the ETEC challenge, we detected the mRNA expression of the tight junction proteins zonula occluden-1 (ZO-1) and occludin in the duodenums using qRT-PCR. After the ETEC challenge, the transcript levels of ZO-1 and occludin in the RNA immunized group declined to 53.8% and 44.4% of the unchallenged animals (Fig. 6D). Meanwhile, the ZO-1 and occludin levels in the PBS treated animals decreased to 40.3% and 1% of the unchallenged animals, respectively (Fig. 6D). Consistent with histopathological characterization, the ETEC challenge damaged the tight junctions of the small intestines in the RNA and PBS treated mice. However, the expressions of ZO-1 and occludin in the RNA immunized animals were either higher or significantly higher than those in the PBS control group, thus indicating that the ETEC total RNA immunization had positive effects on protecting the tight junction structure against the ETEC challenge (Fig. 6D).

## Discussion

This study observed that both active ETEC bacteria and ETEC total RNA could stimulate the inflammation reactions of IPEC cells, thereby inducing the secretion of IL-1β. Besides, serum IL-1β and ETEC specific immunoglobulins were significantly promoted in the mouse model administrated with ETEC total RNA orally or intramuscularly. The ETEC total RNA also augmented strong mucosal immunity by elevating secretory anti-K88ac IgA in the intestinal fluid. The RNA induced humoral and mucosal immunity played a role in protecting the animals against the ETEC challenge. Furthermore, the protective efficacy of the ETEC total RNA positively correlated with its dosage. Our results proved that the ETEC total RNA exhibited immunogenicity to induce K88ac specific antibodies. Supplementing with the ETEC total RNA as an adjuvant significantly improved the protective efficacy of the inactivated ETEC vaccine.

Microbial RNA could be recognized by the host innate immune system through the PRRs, such as Toll‐like receptors (TLRs), retinoic acid inducible gene‐I (RIG‐I)–like receptors (RLRs), and nucleotide-binding oligomerization domain–like receptors (NLRs)^13^. Many previous studies have discovered that viral and bacterial RNA could stimulate host innate immunity. For example, RNA derived from the porcine reproductive respiratory syndrome virus (PRRSV) triggers the production of IL-18 and IL-1β in porcine alveolar macrophages^14^. Reoviral dsRNA stimulated IL-18 and IL-1β secretion in human macrophages^15^. Both Mitoma et al. and Sander et al. reported that the total RNA of nonpathogenic *E. coli* K12 (strain DH5α) could activate the IL-1β production of human and murine macrophages through the NLRP3 inflammasome pathway^15,17^. In this study, we detected that the total RNA of a pathogenic ETEC K88ac strain stimulated the IL-1β responses in the porcine intestinal epithelial cells and a mouse model. While Loss et al. found that the ETEC (K91: K88) stimulation induced IL-8 secretion rather than IL-1β^18^. In our experiment, an MOI lower than 400 was not able to induce IL-1β secretion (data not shown). Therefore, the stimulation of distinct inflammatory factors might be affected by bacterial strain types or inoculation concentrations. However, the specific host PRRs that recognize ETEC RNA require further identification, and the downstream signaling pathways to activate the secretion of proinflammatory factor IL-1β also need to be characterized.

Several previous studies have indicated that pathogen RNA is a potential stimulus that may trigger animal humoral immunity. Martinez-Gil et al. reported the Sendai virus RNA enhanced specific IgG responses in mice co-administrated with influenza A vaccine^16^. Sander et al. discovered that live nonpathogenic *E. coli* significantly induced higher humoral responses than heat-killed *E. coli* (RNA degraded). While supplementing heat-killed *E. coli* with isolated *E. coli* RNA significantly increased serum IgG1 and IgG2b^17^. All the studies above revealed that RNA is a critical factor in stimulating the production of immunoglobulin subclass IgG for humoral immunity. Coherently, several studies have reported increased IgG responses to ETEC or ETEC heat-labile toxin^19,20^. In the present study, we further proved that ETEC RNA alone could induce serum specific IgG in our mouse model. Studies Sander et al. and Barbet et al. did not show any differences in serum IgM induction between mice immunized with live *E. coli* and those with heat-killed *E. coli*, thereby suggesting that *E. coli* RNA is not necessary for IgM induction^17,21^. However, we have detected a significant level of circulating IgM in the animals immunized with ETEC total RNA. It indicated that ETEC RNA alone is sufficient to induce IgM production. IgM is the first antibody produced during the initial response to an antigen and usually reaches peak titers within a month after exposure. Nadeau et al. and Fairbrother et al. reported significant serum anti*-*K88 IgA and IgM levels of the piglets vaccinated with two specific vaccines at 21 dpi, resulting in effective protection against the ETEC challenge^7,22^. Our results also suggested that ETEC total RNA administration enhanced the serum IgA and IgM productions at 20 dpi. Like the reported ETEC vaccines, the K88ac total RNA was also capable of inducing a 20-day duration of protection.

ETEC colonize the mucosal surfaces of the small intestine using their fimbrial antigens. Therefore, ETEC vaccines are developed to obtain mucosal immunities against ETEC invasion. Secretory IgA was detected in porcine intestinal tissues immunized with ETEC fimbriae vaccine^23^. SLA-SE, the agonist of a well-characterized PRRs TLR4, was found to enhance murine intestinal IgA responses of an ETEC recombinant vaccine as an adjuvant^24^. Coherently, we detected a notable increase of ETEC K88 specific IgA level in the intestinal fluid of mice received ETEC total RNA via IM and OR, indicating the ETEC RNA itself delivered by either route could promote the protective immunity at mucosal surfaces. Our histological studies revealed that the murine intestinal structure was damaged after the ETEC challenge in the ETEC RNA administrated mice. Since the mice experienced weight loss right after the ETEC challenge, one possible explanation could be the bacteria colonized the intestinal mucosa and caused the damage, but the mucosal immunity finally protected the animal against the infection. However, we observed an obvious inflammation symptom at 18 h post-ETEC RNA immunization during the murine intestine dissection (data not shown). Moreover, intestinal inflammation caused by ETEC K88 infection and K88 fimbriae immunization has been reported in piglets^23^. Thus, we could not exclude the possibility that the intestinal damage might be due to the inflammation caused by the RNA immunization rather than the ETEC challenge.

T-helper cell differentiation into distinct subtypes plays a role in mucosal immunity through maintaining the equilibrium between antigen responsiveness and tolerance^25^. T-cells can be divided into Th1 and Th2 cells. Th1 cells secrete cytokines such as IL-2, IFN-γ, and TNF-β to trigger cell-mediated immunity. In contrast, Th2 cells produce IL-4, IL-5, and IL-10 to evoke humoral immune responses. Since IFN-γ and IL-4 are mutually antagonistic, the IFN-γ/IL-4 ratio could represent the Th1/Th2 balance^26^. Delisle et al. found enhanced IFN-γ and IL-4 mRNA expression in the intestinal tissue of weaned piglets orally immunized with ETEC K88 fimbriae that adjuvanted with CpG^27^. In line with this, we detected elevated serum levels of both IFN-γ and IL-4 by ELISA in the mice received ETEC RNA administration. Th1/Th2 balance could be influenced by many aspects, such as antigen types, antigen dose, and cytokines^28^. Lee et al. reported that mice vaccinated with F4 fimbriae that orally delivered with thiolated eudragit microsphere (TEMS) triggered similar levels of IgG1 and IgG2a in mice, indicating a mixed Th1 and Th2 immune responses^29^. While the BALB/c mice orally immunized with a live attenuated ETEC vaccine that express fusion enterotoxins induced a Th2 type response^30^. Our results suggested that delivery methods affected the Th1/Th2 bias in the immune responses to the ETEC total RNA. The ETEC RNA administered by the intramuscular injection resulted in a stronger Th2 cytokine response. While the ETEC RNA administered by the oral route slightly increased the IFN-γ/IL-4 ratio compared to the control group, indicating a shift toward Th1 response.

The immunostimulatory activity of pathogen RNA is usually used to develop adjuvants to enhance vaccine effectiveness. For example, RNA derived from Sendai virus significantly improved the performance of an influenza A virus inactivated vaccine as an adjuvant^16^. When supplementing as an adjuvant, nonpathogenic *E. coli* total RNA strongly increased the immunity levels of the mice administrated with heat-inactivated *E. coli*^17^. In previous studies, TLR agonists and attenuated bacterial toxins have been widely used as ETEC vaccine adjuvants to promote protection efficacy^24,31^. Whereas the role of ETEC total RNA as an adjuvant has not been evaluated yet. For this purpose, we administrated a group of mice with 8 μL ETEC vaccine plus 70 μg ETEC RNA as an adjuvant. Due to the suboptimal protective level, the 8 μL dose of the vaccine was selected to reflect the function of the RNA adjuvant. Compared to the 12.5% survival rate in the vaccine administration group, the inclusion of ETEC RNA elevated the survival rate to 75% in the vaccine/RNA mixture immunized group. The ETEC total RNA administration either by IM or OR protected the mice against the ETEC K88 challenge with a survival rate of 75% and 87.5%, respectively. The protective efficiency of immunization with other bacterial RNA has not yet been reported. We immunized the animals with a single dose, but a booster vaccination may improve the protective efficacy. Martinez-Gil et al. reported that mice immunized with an in vitro-transcribed Sendai virus RNA as adjuvant 100% survived against an influenza virus challenge^16^. It reveals that in vitro synthesized RNA with the specific pathogen-origin sequence achieved high protective efficiency, thereby indicating that antigenic RNA has the potential to be manipulated and utilized in vaccine adjuvant development.

We need to point out that 70 μg RNA was a large dose to achieve the desired protection. Synthetic virus RNA can produce high protective effects at a small amount when used as vaccine adjuvants^16^. The composition of viral RNA is relatively simple compared to the bacterial RNA, which is composed of various types. Interestingly, it is reported that different types of *E. coli* RNA trigger the host immunities differently. All three types of *E. coli* DH5α RNA (mRNA, tRNA, and rRNA) could stimulate IL-1β responses in human macrophages via NLRP3 inflammasome activation^17^. Two studies reported that only *E. coli* DH5α derived mRNA could activate the Nlrp3 inflammasome in murine macrophages, but tRNA and rRNAs did not^17,32^. There is no evidence it is the same case for the pathogenic ETEC RNA. But rRNA, which comprises about 80% of the total RNA, seems to critically affect the protection efficiency. In order to improve the immunostimulatory responses of the ETEC RNA, the protective effect of different ETEC RNA types either in the mouse model or in the piglets need to be further studied.

## Materials and methods

### Animal ethics statement

The animal experiment in this study was approved by the Animal Ethical and Welfare Committee (AEWC) of the College of Veterinary Medicine at Hebei Agricultural University (approval number: 2018-12). The animals were treated in strict adherence to the recommendations in the China National Institute of Health guidelines.

### Cell lines and bacterial strains

The IPEC-J2 cells were purchased from Guangzhou Jennio Biological Company (Guangzhou, China). The enterotoxigenic *E. coli* strain (O147: K88ac) and *Salmonella* strain isolated from diarrheic piglets were obtained from the China Veterinary Culture Collection Center (Beijing, China). ETEC K88ac strain was confirmed with PCR amplification of the K88ac fimbriae gene.

### Infection and stimulation of IPEC-J2 cells

The IPEC-J2 cells were infected at a multiplicity of infection (MOI) of 400 for 24 h. For the positive control, IPEC-J2 cells were stimulated with 1 μg/mL lipopolysaccharide (LPS) for 12 h, followed by adding 5 mM ATP for another 5 h of incubation. The ETEC total RNA was isolated using a Bacteria RNA Extraction Kit (Vazyme, China) according to the manufacturer’s instructions. The IPEC-J2 cells were stimulated with 40 μg ETEC total RNA delivered by lipofectamine 2000 (Invitrogen, USA) for 8 h. For the negative control, the same amount of total RNA was treated with RNase A (Solarbio, China) following manufacturer’s instructions. The cells were also treated with the same volume of lipofectamine 2000. The culture supernatants were then collected for the detection of IL-1β using Enzyme-Linked Immunosorbent Assay (ELISA) Kit for Interleukin Beta (Cloud-Clone Corp., USA).

### Mice immunization, sample collection, assessment of immunoglobulins and cytokines

The specific pathogen-free (SPF) BALB/c mice (6-week-old male pups) were purchased from SPF (Beijing) Biotechnology Co., Ltd. (Beijing, China). The Inactivated *E. coli* Trivalent Vaccine for Newborn Piglets against ETEC K88, K99 and 987P strains was purchased from Shandong Huahong Biological Engineering Co. (Shandong, China). This ETEC vaccine contains K88, K99 and 987P antigens (K88 antigen ≥ 100 units/mL, K99 antigen ≥ 50 units/mL, 987P antigen ≥ 50 units/mL). The 64 mice were divided randomly into eight groups (n = 8), immunized with (i) PBS, (ii) 30 μg ETEC total RNA, (iii) 50 μg ETEC total RNA, (iv) 70 μg ETEC total RNA, (v) 8 μL ETEC vaccine, (vi) 40 μL ETEC vaccine, (vii) 70 μg ETEC total RNA treated with RNase A, (viii) 70 μg *Salmonella* total RNA via intramuscular injection. The ETEC vaccine and the RNA were all prepared in PBS with a final volume of 100 μL. The ETEC total RNA was treated with RNase A (Solarbio, China) following the manufacturer’s instructions. A ninth group of mice (n = 8) was immunized with 70 μg ETEC total RNA via oral gavage. The whole blood samples were collected from the submandibular vein at 20 days post-immunization (dpi), respectively. The whole blood was left at room temperature for 2 h, followed by 4 °C overnight, then centrifuged at 4 °C for 20 min at 1200×*g* to separate the serum. The intestinal fluid was collected by flushing the small intestines with 2 mL of PBS, followed by centrifugation at 3000×*g* for 10 min at 4 °C to collect the supernatant. The concentration of serum IL-1β was detected using a RayBio Mouse IL-1β ELISA Kit (Raybiotech, USA). K88ac specific antibodies (IgG, IgM, and IgA) were evaluated by ELISA using commercial kits purchased from Jiangsu Meimian Industrial Co., China. IL-4 and IFN-γ were assessed by ELISA using commercial kits purchased from USCN Life Sciences, China.

### ETEC challenge

For the ETEC challenge, 64 BALB/c mice were randomly divided into eight groups (n = 8), immunized with (i) 30 μg ETEC total RNA, (ii) 50 μg ETEC total RNA, (iii) 70 μg ETEC total RNA, (iv) 8 μL ETEC vaccine, (v) PBS, (vi) 8 μL ETEC vaccine plus 70 μg ETEC total RNA, (vii) 70 μg ETEC total RNA treated with RNase A, (viii) 70 μg *Salmonella* total RNA via intramuscular injection. The ETEC vaccine and ETEC total RNA were all prepared in PBS with a final volume of 100 μL. A ninth group of mice (n = 8) was immunized with 70 μg ETEC total RNA via the oral route. At 23 dpi, all groups were challenged with 3 × 10^8^ CFU of ETEC via intraperitoneal injection. The survival and weight loss were monitored for 96 h post-challenge. A tenth group was used as a positive control, which remained unimmunized and unchallenged. The duodenum samples of the mice from the 70 μg RNA immunized group, PBS group, and positive control group were collected at 96 h post-challenge, rapidly frozen in liquid nitrogen, and stored at − 80 °C for further experiments.

### Hematoxylin and eosin staining

The duodenum samples were immersed in formalin fixation solution and sent to Wuhan Cloud-Clone Co. (Hubei, China) for embedding into paraffin blocks. The paraffin sections were sliced using a Rotary Microtome (Hestion, China). The sliced sections were stained using a Hematoxylin–Eosin/HE Staining Kit (Solarbio, China), sealed with neutral resin, and observed under a microscope (Olympus, Japan).

### RNA isolation from mouse small intestine and qRT-PCR

The duodenum samples plus TRIzol reagent (3 mL/100 mg) (Invitrogen, USA) were ground with a glass tissue homogenizer, followed by standard procedures of RNA isolation via TRIzol reagent. The cDNA was synthesized using HiScript II 1st Strand cDNA Synthesis Kit (Vazyme, China), then qRT-PCR was carried out with iQ SYBR Green Supermix (Bio-Rad, USA). The primers of occludin, ZO-1, and GAPDH used in qRT-PCR are listed in Table 2.

**Table 2:** Primer sequences of quantitative real-time PCR.

Gene symbol	NCBI reference sequence	Primer sequences	Product size
Occludin	NM_008756.2	F^a^: CACCCCCATCTGACTATGCG	120 bp
		R^b^: CAGGCACCAGAGGTGTTGAC	
ZO-1	NM_009386.2	F: ACAGCTACAGGAAAATGACCGA	180 bp
		R: TTATCAGACACCGGCTCAGG	
GAPDH	NM_001289726.1	F: AACGTGTCGGTTGTGGATCT	140 bp
		R: TCACAGGACACAACCTGGTC	

^a^Forward primer.

^b^Reverse primer.

### Statistical analysis

Statistical analyses were performed using Student’s *t*-test via the IBM SPSS Statistics Version 19 (https://www.ibm.com/products/spss-statistics). The data are presented as mean ± SD for each experiment. *P* ≤ 0.05 was considered statistically significant. All of the experiments were performed in triplicate.

## Data Availability

The authors declare that all of the available data are present in the manuscript.
